# Mom feels what her child feels: thermal signatures of vicarious autonomic response while watching children in a stressful situation

**DOI:** 10.3389/fnhum.2013.00299

**Published:** 2013-06-25

**Authors:** Barbara Manini, Daniela Cardone, Sjoerd J. H. Ebisch, Daniela Bafunno, Tiziana Aureli, Arcangelo Merla

**Affiliations:** ^1^Infrared Imaging Lab, Institute of Advanced Biomedical Technologies, G. d'Annunzio UniversityChieti-Pescara, Italy; ^2^Department of Neuroscience and Imaging, G. d'Annunzio UniversityChieti-Pescara, Italy

**Keywords:** emotion, vicarious responses, emotional sharing, mother-child synchrony, mother-child bond, autonomous nervous system, IR thermal imaging

## Abstract

Maternal attunement with an infant's emotional states is thought to represent a distinctive feature of the human primary bond. It implies the mother's ability of empathizing with her child in order to fulfil the child's needs in an immediate and appropriate manner. Thus, it is particularly involved in stressful situations. By assuming that maternal attunement embodies a direct sharing of physiological responses with the child, we compared the autonomic response of mothers observing their own distressed child with those of other women observing an unknown child involved in an ecological distressful condition (mishap paradigm). The hypothesis was that the adult's response was more attuned with the child's response in the former group than in the latter group. The autonomic response was non-invasively evaluated through the recording of the thermal facial imprints by means of thermal infrared (IR) imaging. Nine mother-child dyads and 9 woman-unknown child dyads were studied. We found marked similarities between the facial temperature dynamics of women and children along the experimental procedure, thus providing evidence for a direct emotional sharing within the adult-child dyad. The evidence for common dynamics in the time course of the temperatures was assessed through correlation analysis and, nevertheless, resulted stronger in the mother-child dyads than in the other women-child dyads. In addition, temporal analysis showed a faster response in mothers than in other women, thus confirming our study hypothesis. Besides confirming the extraordinary capability of IR imaging to preserve ecological context in the study of social or non-verbal interactions, these results suggest that maternity appears to potentiate the emotional attunement with the child. Although based on preliminary results, this study opens new perspectives in the study of the factors modulating vicarious socio-emotional processes.

## Introduction

Maternal attunement with a child is a key element of secure attachment relationships (Bowlby, 1958). A mother who is capable to share affect with her child, to empathize with his/her emotional needs and to appropriately respond to his/her requests, allows the infant to perceive a sense of being accepted and recognized, which facilitates social adjustment and a positive psychological functioning (Bowlby, 1958). Conversely, the lack of such a sensitivity could lead to less favorable outcomes in the child's subsequent development (Sullivan et al., 2011). Although there is a general agreement on the importance of maternal attunement, little is still known about the biological basis of that ability.

Maternal attunement with offspring is especially involved when mothers are facing infants' distress. As shown by a large amount of empirical evidence, mother's alertness and arousal increase because of the baby's distress signals (Swain et al., 2007), allowing to immediately provide the help needed for the infant's recovery (Bell and Ainsworth, 1972; Frodi and Lamb, 1978; Mills-Koonce et al., 2007). As found by neuroimaging research, watching a child's emotional expressions specifically activates some brain regions, like the anterior insula, the amygdala and the mirror neuron system (Rizzolatti et al., 2001; Gallese et al., 2004). This activation occurs mostly when a mother observes her own child rather than an unknown child (Iacoboni et al., 1999; Lenzi et al., 2008). Since the anterior insula is considered the relay between the action representation (mirror neuron system) and the emotional processing (limbic system) (Lenzi et al., 2008), the above activation could relate to the mother understands of her own child's distress signal, probably to the aim of responding successfully and appropriately. Particularly, the infant's crying—the most powerful distress signal—activates the same brain regions involved in attention, emotional attachment and in the process of integrating autonomic states with social behavior (Seifritz et al., 2003; Swain et al., 2007, 2008; Kim et al., 2010; Swain, 2011; Swain et al., 2011). The infant's distress also elicits autonomic responses in the own mother. In particular, blood pressure as index of aversive stimuli, heart rate as attentional and defensive response, and skin conductance as index of arousal, increase in mothers witnessing their infant's crying (Frodi and Lamb, 1978).

Beyond the ability to provide the right intervention in distressing situations, synchronization of the mother's responses to the infant's signals in typical dyadic interactions can be considered a key aspect of sensitive parenting, as it implies the promptness of the mother response (Bell and Ainsworth, 1972) and the adaptation moment by moment to the child's emotional states (Noriuchi et al., 2008; Guedeney et al., 2011). Repeated experience of well-synchronized and appropriate interactions allow mother and child to become sensitive to each other's physiological and behavioral cues (Fleming et al., 1999; Feldman, 2007; Feldman et al., 2011, 2012) and capable to perceive the other's behaviors as a response to their own behavior, which contributes to the formation of a unique bond between them (Stern, 1985; Mogi et al., 2011).

The autonomic nervous system seems to represent an elementary mechanism supporting emotional synchrony between mother and infant. According to Porges (1998, 2003a,b), the emergence of parenting behavior in mammals is linked to the development of the Polyvagal system (Feldman and Eidelman, 2007). In his opinion and with reference to the human realm, the decrease of heart-rate variability due to vagal-tone suppression in response to a stressful event (Porges, 2003a,b) would facilitate complex behaviors such as attention, orientation and the maintenance of calm states, which are required for early formation of secure social bonds and for achieving the more advanced coordination of social signals that underlie human interpersonal interactions. Supporting that hypothesis, research on early infancy found that the mother's and her child's vagal tone are inter-related (Feldman et al., 2010), thus suggesting that the degree of parasympathetic control during social engagement is shaped by co-regulatory processes. As suggested by Feldman (2007); Feldman et al. (2012), biological synchrony in interpersonal interactions shows online sensitivity to the partners' ongoing behavior.

The physiological side of the mother-infant bond has been typically observed in stressful situations. Mother's and infant's cortisol reactivity were found to be strictly associated in the still-face paradigm, an interactive situation eliciting some degree of stress in infants (Haley and Stansbury, 2003; Feldman et al., 2010), and while playing a challenging- for-the-child game (Sethre-Hofstad et al., 2002). All together, these findings provide prototypical examples of the bio-behavioral synchrony underlying interactions, as suggested by Feldman (2007). Accordingly, an autonomic-visceral synchrony between mother and child was found in our previous study (Ebisch et al., 2012), showing a significant parallelism between mothers' and children's facial temperature variations when mothers observed their own children involved in a stressful situation. Since skin temperature is mediated by the autonomous nervous system and varies in response to emotional stimuli originated from external environment (Anbar, 2002; Merla and Romani, 2007; Nakanishi and Imai-Matsumura, 2008; Shastri et al., 2009; Nhan and Chau, 2010; Kuraoka and Nakamura, 2011), that study showed for the first time a direct sharing at the autonomic level between mother and child associated with an experience of affective attunement.

The present study aimed to deepen the results found by our previous one (Ebisch et al., 2012). In that study we demonstrated autonomic thermal synchrony in mother-child dyads when the mother watched her own child experiencing a stressful situation elicited by the mishap paradigm (Cole et al., 1992). Thermal autonomic responses were assessed through the use of high-resolution thermal infrared (IR) imaging (Merla and Romani, 2007). In the present study, using the same paradigm of the previous study, we compared the thermal autonomic responses between two groups of dyads: mother-her own child dyads and women-unknown child. Since, according to previous research (Seifritz et al., 2003; Swain et al., 2007, 2008), the experience of one's own child distress seems to influence the women's reactions to the infant's emotional needs, we expected to find different autonomic responses, on either the intensity or the time scale, across the groups. Specifically, we hypothesized that the former group of dyads would attune more than the second group, thus showing a stronger shared response. Alternatively, the two groups could exhibit the same physiological response, thus suggesting that the such a response to the child's distress is a general reaction, independently of the specific bond with the distressed child. Moreover, according to evidence for the contingency of maternal responses as one of the specific characteristics of maternal sensitivity (Feldman, 2007; Feldman et al., 2012), we expected to find more ready and prompt responses in the first group of dyads.

## Methods and materials

### Participants

Fourteen children (seven male, age 39–45 months) participated in the study. All of the children were born at term and had a typical psychological and physical development. The adult sample was composed of eighteen women divided in two groups: the first one included nine mothers (age 25–43) who watched their own child during the experiment (“mothers,” M); the second sample was composed of 9 women (age 23–38) (“other woman,” OW). Four of these had preschool children not participating to the study, while the other five were not mother. The data about six out of nine mother-child dyads were included in our previous study (Ebisch et al., 2012). The two groups of women were matched for socio-economical status and study degree. Inclusion criterion for adult subjects was the absence of any overt physical, psychiatric or psychological disease. All participants were asked to refrain from heavy physical activities and consumption of coffee, cigarettes and vasoactive substances for at least 2 h prior to the measurements, and to avoid cosmetics on their faces at the moment of the experiment. The study was approved by the Local Ethics Committee. Written informed consent was obtained from all the participants in agreement with the Declaration of Helsinki.

### Procedure

Prior to testing, each subject was left to acclimatize for 10–20 min to the experimental room and to allow the neutral condition skin temperature to stabilize. The recording rooms were set at standardized temperature (23°C), humidity (50–60%) without direct ventilation. The subjects sat comfortably on a chair during both the acclimatization and the measurement periods, without any restriction of body movements. Before the start of the experiment, the children underwent an adequate familiarization period to ease the psychological habituation to the setting and the experimenter, first in presence of their mothers, then followed by neutral interaction with the experimenter alone. During neutral interaction between the experimenter and the child, some toys were presented to allow the child to feel at ease and to get used to play with the experimenter.

After the neutral interaction with the experimenter, the children were exposed to a potential stressful experience, elicited by the “Mishap Paradigm” (Cole et al., 1992). More specifically, children were invited to play with a toy, which was previously manipulated to break in the child's hands when playing with it, thus suggesting that the child accidentally broke the toy. The toy was introduced by the experimenter as her own favorite. Distinct phases could be distinguished in the paradigm: (1) “presentation” (the experimenter demonstrated the toy); (2) “playing” (the child played with the toy, while the experimenter left the room for 1 min); (3) “mishap” (child “broke” the toy); (4) “re-entry” of the experimenter (the experimenter did not say anything for 30 s and merely looked at the broken toy); (5) “soothing” of the child (the experimenter cheerfully indicated that the toy could be fixed and that the breaking was not the child's fault).

In order to perform the analyses, the above mentioned phases were grouped in conditions. The neutral interaction defined the “neutral condition”; the presentation and playing phases together formed the “intermediate condition”; the mishap, re-entry and soothing phases together formed the “experimental condition.”

The “mothers” and the “other women,” naive about the specific content of the experiment, were invited to silently observe the children-experimenter interaction through a one-way mirror from a separated room. It was possible that two women (one for each group of dyads) watched at the same child together, though they could not see each other or interact as a screen was placed between them.

Facial thermal images for all of the subjects were recorded along the whole experimental procedure (acclimatization, neutral, intermediate, and experimental conditions).

### Materials

Thermal IR imaging was performed by means of three digital thermal cameras FLIR SC660 (640 × 480 bolometer FPA, sensitivity: <30 mK @ 30°C). The acquisition frame rate was set to 15 Hz for each thermal camera.

Two remote-controlled video-cameras (Canon Vc-C50iR) were used to film the child for the behavioral analysis. Video-signals were sent to two video-recorders (BR-JVC) and mixed by a Pinnacle system (Liquid 6). Subsequently, the movies were processed through a specialized software (Interact Plus, Mangold) that allows to code behavior in synchrony with the ongoing movies of the children during the experiment. The toy presented to the children in the “Mishap Paradigm” was a black and white robot with a height of approximately 20 cm. When turning on the robot with a switch on its back, it started to walk and play music. Both hands of the robot could be opened and closed by means of pressing/relieving a button. One of the hands of the robot were prepared to break when manipulated by the child. The robot could be repaired only by the experimenter. The toys presented during neutral interaction between the experimenter and the child were a puzzle, a magic wand and 3-D book.

## Data analysis

### Behavioral data analysis

Following from the notion that different combinations of relevant signs in a mishap situation may be indicative of guilt (Barrett et al., 1993; Kochanska et al., 1995), we coded the presence or absence of the child's reactions into five categories (see Table 1): *gaze and eye*, *bodily tension*, *arms*, *repair*, *and verbalizations* (Barrett et al., 1993; Kochanska et al., 2002; Mills, 2003; Barrett, 2005). The duration of the phases was similar among children [*M* = 35.86 (*SD* = 17.54) seconds in playing; *M* = 62.21 (*SD* = 14.52) seconds in mishap; *M* = 48.86 (*SD* = 16.44) seconds in re-entry], and there were no cases of distress so important to reduce the duration of the phase in a relevant way for the appearance of the behaviors to code. Thus, in playing, mishap and re-entry of experimenter, overall distress response was coded on a 4-point scale [(1) child was not affected in any way; (2) child appears middle distressed as evidenced by one or two signal coded; (3) child appear distressed as evidenced by three or four signal coded; (4) child is strongly distressed as evidenced by five signal coded]. Behaviors were coded with the software INTERACT 8.

**Table 1:** **Expressive features coded for the scoring of child reactions**.

**Category**	**Features**	**Description**
Gaze and face^a^^,^^b^	Gaze aversion	The child stares into space, or toward the oblique low, or toward another insignificant object (excluding the broken toy and the experimenter).
	Lip rolled-in	Lower lip rolled-in; corners of mouth drawn.
Bodily tension^b^	Bodily avoidance	The child backs up while looking at the experimenter; or moves away from her, toward insignificant object, after focusing on her.
	Hunched shoulders	Relaxed or hunched shoulders.
	Head lowered	Head hanging or tilted forward.
Arms^b^	Arms across body	Arms across the midline, held close to the body (e.g., hugging the body).
	Covering, touching face	The child covers or touches all or part of the face.
	Fingers in mouth	Putting a finger or fingers in mouth.
Repair^c^	Trying to repair the object	The child tries to repair, to fix the toy or he/she manipulates it. It is not coded as repair if the action is meant to play.
Verbalizations^b^^,^^c^	Confession	The child admits to have broken the toy e.g., saying “I broke it” or “I pulled this piece off.”
	Negative self-evaluation	The child judges him/herself negatively, e.g., saying “I am not able to play” or “I can't do it.”

a *Barrett et al., 1993; Barrett, 2005*.

b *Mills, 2003*.

c *Tangney and Fischer, 1995; Stipek, 1995; Lewis et al., 1998; Kochanska et al., 2002*.

Comparisons were made between the playing, mishap and re-entry phases, because in these phases the children were engaged with the same object and that made more reliable the coding of their behaviors.

#### Reliability

Two observers coded all the children for each phase and Kappa's ranged from 0.72 to 0.80 (all *p* < 0.05).

### Thermal data analysis

A visual inspection of the changes in facial thermal imprints in all subjects was performed to qualitatively investigate the autonomic responses of the women and the children throughout the experiment. This analysis was followed by a quantitative estimation of temperature variations of the nasal tip. Referring to the literature about thermal signature of emotions (Shastri et al., 2009; Nhan and Chau, 2010; Kuraoka and Nakamura, 2011), the nasal tip has been proved to be associated with the activation of the sympathetic nervous system by emotional and distressing stimuli (Roddie, 1963; Merla and Romani, 2007; Nakanishi and Imai-Matsumura, 2008; Shastri et al., 2009; Nhan and Chau, 2010; Kuraoka and Nakamura, 2011; Ebisch et al., 2012). More precisely, temperature decrease of the nasal tip is related to sympathetic alpha-adrenergic vasoconstrictor activity and the reduction of this vasomotor effect is related to the thermal recover due to the vasodilatation effect.

Thermal signals have been extracted through the use of tracking software, developed with homemade Matlab algorithms (The Mathworks Inc., Natick, MA). The tracking algorithm is based on the 2-D cross-correlation between a template region, chosen by the user on the initial frame, and a similar ROI in a wider searching region, expected to contain the desired template in each of the following frames (Tangherlini et al., 2006). In this way it is possible to automatically extract the thermal signals in defined regions of interest during the whole experiment. The extracted thermal data have been filtered subsequently with a low-pass filter (*f*_cut−off_ = 0.2 Hz), to eliminate breathing effects (Ebisch et al., 2012).

A comparison was made between the neutral, the intermediate, and the experimental conditions. Because the mishap paradigm is an ecological situation the duration of the conditions depended on the children spontaneous behaviors. Therefore, the timing of the experiment presented some variations within subject. The mean duration of neutral condition was 134.62 (*SD* = 80.21) s. The mean duration of the intermediate condition was 139.104 (*SD* = 115.097) s. For the experimental condition the mean duration was 222.327 (*SD* = 55.79) s.

### Statistical data analysis: individual dyads

The thermal signals of the nasal tip for all the subjects were transformed in *z*-scores. A repeated measures (3 × 3) ANOVA was performed on the mean nasal tip temperature of the three conditions for all subjects. The conditions (neutral, intermediate, and experimental) were set as within-subject factor. The groups (children, mother, other women) were set as between-subject factor. Pairwise multiple comparisons between conditions were adjusted for multiple comparisons by means of Bonferroni correction. The goal of this analysis was to control the autonomic response of the subjects during the entire paradigm. In particular our hypothesis was that there were no differences in autonomic response of the subjects between neutral and intermediate condition. On the contrary, it was hypothesized that the autonomic response in the experimental condition was significantly different, compared with both the neutral and the intermediate condition. Besides, we wanted to investigate if this autonomic response changed according to the specific group of the subjects. Pearson correlation coefficients among the time courses of the temperature signals were calculated for each women-child dyad and for the neutral and the experimental condition. Correlation coefficients were then transformed according to Fisher's distribution (*r* to *z* transform) and a *t*-test was performed to evaluate whether significant differences could be found between conditions (neutral and experimental) and groups of dyads in the two conditions.

A cross-correlation like analysis on each dyad's temperature signals during the experimental condition was also performed to test which delay between the signals corresponded their highest correlation value. For this purpose, Pearson correlation coefficients were computed by delaying the woman's signal with respect to the child's signal with 10, 20, 30, 40 s. For each group, the mean values of the delays corresponding to the highest dyad correlation were computed. A Mann–Whitney *U*-test was performed to assess possible differences between the two groups of dyads regarding the distributions of time delays maximizing the correlations.

As the “other women” group included four mothers, explorative descriptive analyses on the “other women” subgroups were performed to preliminarily investigate possible differences between other mothers and non-mothers. Pearson correlation coefficients were calculated on the individual dyads level for the other mothers and non-mothers subgroups. Furthermore, the temporal modulation of the signal was explored for the two subgroups.

### GROUP analysis

Since the experiment duration depended on the child's behavior, signal resampling was performed to compare the three conditions and the three groups (children, mother, other women). Each temperature signal was resampled in order to obtain 300 data points for each condition, equally spared across the time duration of the condition (for the experimental condition, 120 samples were included for the mishap phase, 60 for the re-entry phase and 120 for the soothing phase). The resampled *z*-score temperatures where then averaged for the groups, thus providing an average time course for each group and condition.

## Results

### Behavioral results

Behavioral analyses showed that all children experienced an increase of distress in response to the mishap. Specifically, the repeated-measures ANOVA indicated that the children reacted in a different way in the playing, mishap and re-entry of experimenter phase [*F*_(1, 13)_ = 10.95; *p* < 0.05]. There was an evident increase of distress signs in both the mishap and experimenter re-entry phase, compared with playing (*t* = 4.37, *p* < 0.01; and *t* = 3.31, *p* < 0.01, respectively). No significant evidence of difference in distress signs was found comparing the mishap and the experimenter re-entry phases. Table 2 reports the results of the behavioral analysis.

**Table 2:** **Means and standard deviation of the categories in all phases coded**.

**Categories**	***N***	**Phases**					
		**Playing**		**Mishap**		**Re-entry**	
		**Mean**	***SD***	**Mean**	***SD***	**Mean**	***SD***
Gaze and face	14	0.57	0.51	0.86	0.36	0.71	0.47
Bodily tension	14	0.14	0.36	0.36	0.50	0.50	0.52
Arms	14	0.21	0.42	0.29	0.47	0.29	0.47
Repair	14	0	0	0.64	0.50	0.43	0.51
Verbalizations	14	0	0	0.21	0.43	0.21	0.43
Total scores	14	1.71	0.47	2.42	0.51	2.28	0.61

### Visual analysis of facial thermal imprints

A visual inspection of the facial thermal imprints was performed to investigate the presence of appreciable signs of autonomic responses in the mothers, the other women and the children throughout the experiment. The qualitative results resembled those already found in our previous work (Ebisch et al., 2012).

**Figure 1:**
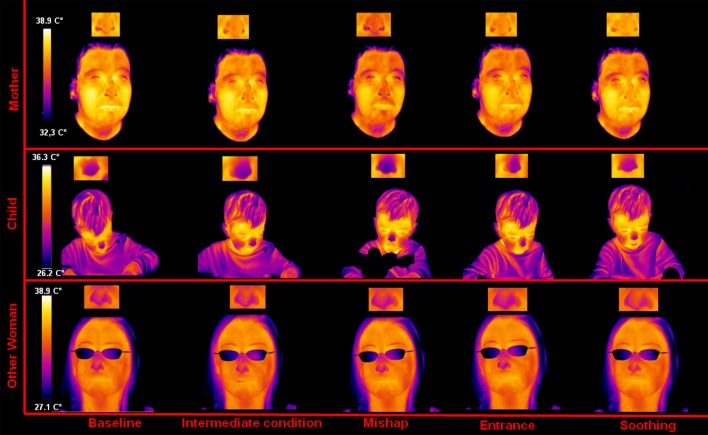
**Facial thermal imprints of one of the mother-other woman-child triads**.

A representative example of the facial thermal imprints of one mother-other woman-child triad is shown in Figure 1. As to the child, no appreciable modulations were detected regarding the facial skin temperature distribution between the neutral condition and the intermediate condition. However, after the mishap a sympathetic reaction could be observed, reflected by a sudden and wide-spread decrease of face temperature, especially in the nasal tip as previously found in human as well as macaques (Kistler et al., 1998; Merla and Romani, 2007; Nakanishi and Imai-Matsumura, 2008; Shastri et al., 2009; Nhan and Chau, 2010; Kuraoka and Nakamura, 2011; Ebisch et al., 2012). The decreased skin temperature in the nasal tip likely reflects peripheral vasoconstriction due to the alpha-adrenergic activity. These sympathetic responses gradually decrease after the re-entry of the experimenter. During the soothing phase, the temperature of the nasal tip soon increased, likely reflecting a withdrawal of the sympathetic alpha-adrenergic vasoconstrictor effect. Moreover, the nasal tip temperature presented an over-response, compared to the neutral condition value.

Concerning the mother, no appreciable modulation of skin temperature distribution was detected during the intermediate condition. After the mishap as well as after the re-entry of the experimenter, the same thermal variations observed in the child could be appreciated in the mother. During the soothing phase, the mother showed a gradual and generalized increase of facial temperature, re-establishing the neutral condition state. Moreover, like the child, she showed an over response of the nasal tip temperature, compared to the start of the experiment.

Finally, the other woman did not show any signal modulation between neutral condition, intermediate condition, and mishap. During the experimenter re-entry, there was a cooling of the nasal tip, however, milder (using the same temperature scale and image contrast) than that showed by the child and the mother. In the soothing phase the other woman showed a recovery of the nasal tip temperature to the initial neutral condition value.

### Comparisons of autonomic responses between groups and conditions

An ANOVA (3 × 3) was performed on the re-sampled *z*-score mean temperatures of the nasal tip. A significant within-subject effect was found for condition [*F*_(2, 29)_ = 5.70; *p* < 0.01]. Bonferroni adjusted pairwise comparisons on the within-subject factor “condition” showed no significant difference between neutral and intermediate condition (*p* = 1). There was a significant difference between the neutral and the experimental condition (*p* < 0.05) and between the intermediate and the experimental condition (*p* < 0.05). The condition × group effect was not significant [*F*_(2, 29)_ = 0.58; *p* = 0.68]. These results suggest that the experimental condition, but not the intermediate condition induced a significant modulation on autonomic response in all three groups (i.e., children, mothers and other women), compared with the neutral condition.

### Correlation analysis

Table 3 shows the Pearson correlation coefficients calculated for all the dyads in the neutral condition and in the experimental condition. As we were interested in studying the attunement of the autonomic responses of the mothers with those evoked by the distressful condition in the child, the non-distressful intermediate condition was not included in the correlation analysis. Pearson correlation coefficients were found to be statistically significant (all *p* < 0.001). With respect to the mother-child dyads group, the mean coefficient value resulted *r*_mean_ = 0.17 (*SD* = 0.60) during the neutral condition and during the experimental condition it increased up to a mean value of *r*_mean_ = 0.70 (*SD* = 0.27). The *t*-test on the Fisher-transformed *r*-values showed a significant difference in the correlation coefficient between neutral condition and experimental condition in the mothers-child dyads group (*t* = −3.32, *df* = 8, *p* < 0.05).

**Table 3:** **Pearson correlation coefficient for all the dyads**.

**Dyad**	**Type of dyad**	**Neutral condition**	**Experimental condition**
1	M-C	0.53^*^	0.67^*^
2	M-C	0.51^*^	0.76^*^
3	M-C	−0.51^*^	0.71^*^
4	M-C	0.49^*^	0.84^*^
5	M-C	0.86^*^	0.91^*^
6	M-C	−0.64^*^	0.94^*^
7	M-C	−0.61^*^	0.28^*^
8	M-C	0.86^*^	0.97^*^
9	M-C	0.06^*^	0.23^*^
10	OW-C	−0.12^*^	0.24^*^
11	OW-C	0.26^*^	0.58^*^
12	OW-C	−0.77^*^	−0.03^*^
13	OW-C	−0.85^*^	0.82^*^
14	OW-C	0.35^*^	−0.38^*^
15	OW-C	−0.24^*^	0.75^*^
16	OW-C	0.29^*^	0.40^*^
17	OW-C	0.93^*^	0.71^*^
18	OW-C	−0.32^*^	0.54^*^

*In the column type of dyad M-C, mother-child dyads; OW-C, other women-child dyads*.

* *p < 0.001*.

With respect to the other woman child-dyads, in 5 out of 9 cases there was an increase of correlation in the experimental condition, compared with the neutral condition. The mean value of the neutral condition correlation was *r*_mean_ = −0.05 (*SD* = 0.57), while the mean value in the experimental condition was *r*_mean_ = 0.40 (*SD* = 0.39). The *t*-test showed no differences between neutral condition and experimental condition in this second group (*t* = −1.64, *df* = 8, *p* = 0.14).

The comparison between the two groups suggested that there was no significant difference regarding correlation coefficients in the neutral condition (*t* = 0.76, *df* = 16, *p* = 0.45). Instead, the *t*-test yielded a significant difference between the two groups in the experimental condition (*t* = 2.19, *df* = 16, *p* < 0.05), reflecting a higher mean correlation in the mother group, compared with the other women group.

Finally, an explorative descriptive analysis was performed in order to assess the differences in correlation coefficient between other mothers-child dyads and non-mother-child dyads belonging to the other women group. The mean correlation in the other mother-child dyads was *r*_mean_ = 0.16 (*SD* = 0.58) for the neutral condition and *r*_mean_ = 0.60 (*SD* = 0.58) for the experimental condition. In the non-mother-child dyads the mean correlation is *r*_mean_ = 0.16 (*SD* = 0.58) for the neutral condition and *r*_mean_ = 0.25 (*SD* = 0.47) for the experimental condition.

### Temporal analysis of the responses

Table 4 shows the Pearson correlation coefficients for the thermal signal of the nose tip of adult and child for the experimental condition, in both the groups of dyads. In order to evaluate whether the maximum value of the correlation varied along the time course of the experimental phase, i.e., to evaluate possible delayed responses, and whether such eventual delays differed between the two groups of dyads, we computed the Pearson correlation coefficients by delaying the woman's signal with respect to the child's one with 10, 20, 30, 40 s, like in a cross-correlation analysis. The largest delay with respect to which we report the cross-correlation results is 40 s, since a higher delay did not maximize the correlation value in any dyads (see Table 4).

**Table 4:** **The table shows the Pearson correlation coefficient for all dyads for synchronous and shifted signals**.

		**Delay**				
**No**.	**Type of dyads**	**No delay**	**10 s**	**20 s**	**30 s**	**40 s**
1	M-C	0.24^*^	0.96^*^	0.94^*^	0.92^*^	0.90^*^
2	M-C	0.28^*^	0.24^*^	0.21^*^	0.19^*^	0.16^*^
3	M-C	0.97^*^	0.34^*^	0.41^*^	0.5^*^	0.58^*^
4	M-C	0.82^*^	–0.34^*^	–0.35^*^	–0.39^*^	–0.29^*^
5	M-C	0.76^*^	0.72^*^	0.94^*^	0.47^*^	0.92^*^
6	M-C	0.67^*^	0.76^*^	0.78^*^	0.83^*^	0.85^*^
7	M-C	0.71^*^	0.63^*^	0.44^*^	0.19^*^	–0.03^*^
8	M-C	0.84^*^	0.74^*^	0.50^*^	0.13^*^	–0.20^*^
9	M-C	0.91^*^	0.89^*^	0.69^*^	0.64^*^	0.69^*^
10	OW-C	0.75^*^	0.79^*^	0.84^*^	0.88^*^	0.91^*^
11	OW-C	0.40^*^	0.30^*^	0.28^*^	0.14^*^	–0.17^*^
12	OW-C	0.71^*^	0.67	0.68^*^	0.62^*^	0.35^*^
13	OW-C	0.51^*^	0.28^*^	0.35^*^	0.45^*^	0.55^*^
14	OW-C	0.24^*^	0.60^*^	0.68^*^	0.71^*^	0.74^*^
15	OW-C	0.58^*^	0.51^*^	0.49^*^	0.49^*^	0.47^*^
16	OW-C	0.28^*^	0.48^*^	0.60^*^	0.68^*^	0.74^*^
17	OW-C	0.55^*^	–0.52^*^	–0.53^*^	–0.52^*^	–0.46^*^
18	OW-C	0.82^*^	0.79^*^	0.86^*^	0.88^*^	0.98^*^

*In the column type of dyad M-C, mother-child dyads; OW-C, other women-child dyads*.

* *p < 0.001. Bold value indicates maximum correlation coefficient for the dyad*.

The average value of the maximum correlation coefficients for the mothers-child dyad group was *r*_mean_ = 0.81 (*SD* = 0.21), corresponding to an average delay of 7.8 (*SD* = 13.94) s.

One mother-child dyad (dyad #6) reached its maximum correlation value at a 40-s delay, even though its correlation coefficient was already larger than 0.67 and significant at 0 time delay. By excluding this dyad from the group, the average value of the highest correlation coefficients did not change, but there was a significant decrease of the mean delay in which *r*-value is maximized in the mother-child dyads. Excluding mother-child dyad number six reduced the average delay to 3.75 s (*SD* = 7.44).

The average value of the maximum correlation coefficients for the other woman-child dyad group was *r*_mean_ = 0.57 (*SD* = 0.42), corresponding to an average delay of 26.7 (*SD* = 20.0) s. The Mann–Whitney *U*-test on the delay levels showed a significant difference between the two groups (*U* = 14, *p* < 0.05), even when taking into account the dyad #6 in the mother-child dyad group.

Although aware of the small size of the sample, an explorative analysis was performed on the other women group, by dividing it in two subgroups: other mothers (i.e., mothers watching not their own child) and non-mothers. The mean of the maximum correlation coefficients in other mother-child dyads was *r*_mean_ = 0.64 (*SD* = 0.21), whereas the mean of the delays was 20 s (*SD* = 23.09). In the non-mother-child dyads the mean of maximum correlation was *r*_mean_ = 0.52 (*SD* = 0.56) with an average delay of 32 s (*SD* = 17.88).

### Group analysis of correlation

**Figure 2:**
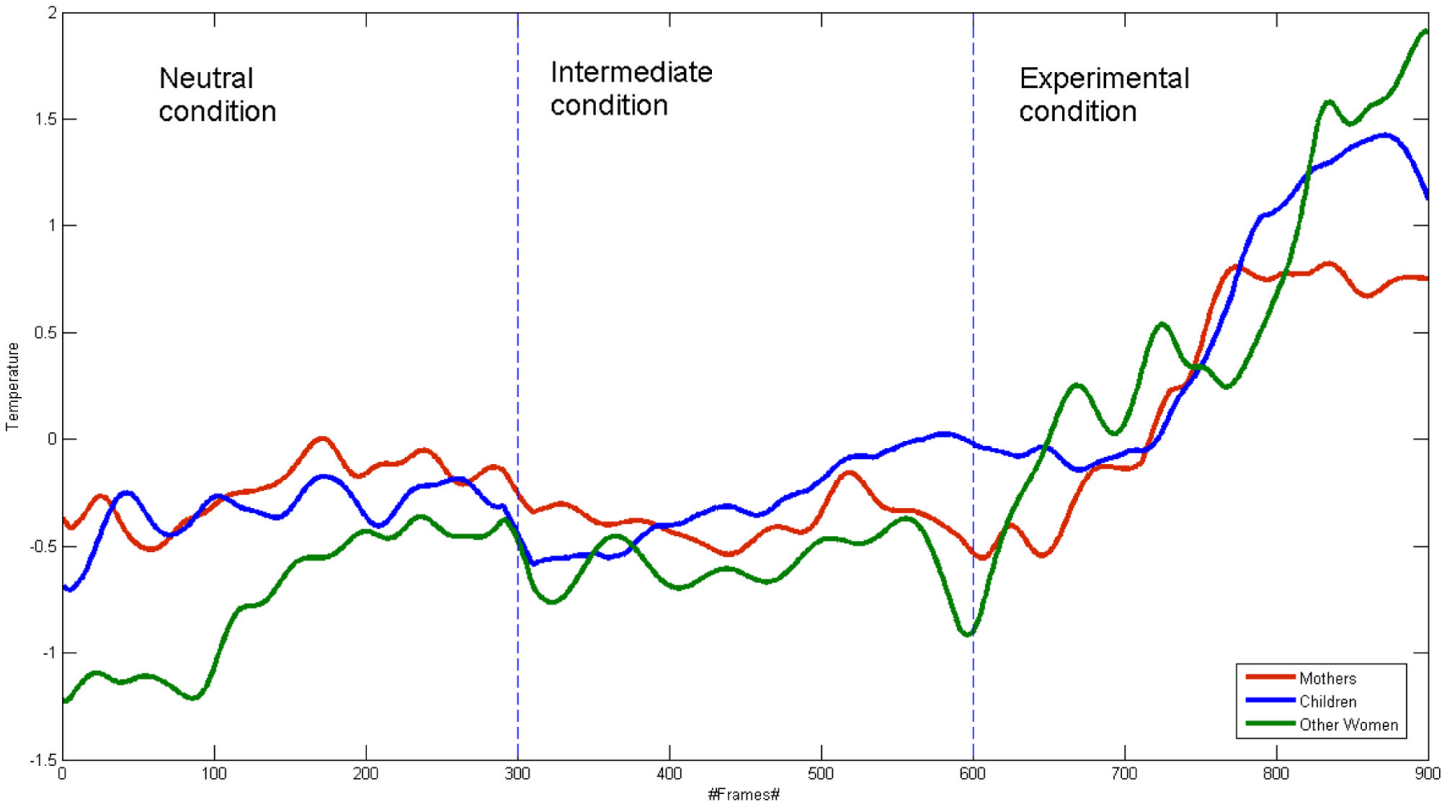
**Mean signals of mothers, other women and children for all the conditions**.

Figure 2 shows the groups' average signal. The correlation coefficients were calculated among the three average signals, for the their whole time course and for each condition. For the whole time course signal, the correlation coefficient value for the mother-child dyads was *r* = 0.88 (*p* < 0.001), while it resulted *r* = 0.84 (*p* < 0.001) for the other women-child dyads. The Pearson coefficient values did not differ between the two groups in the experimental condition (mother—child: *r* = 0.89 *p* < 0.001; other women—child: *r* = 0.90 *p* < 0.001).

## Discussion

The present study focused on the physiological expression of the emotional attunement of mothers to their own children. We compared the autonomic response, measured through the nose tip temperature, of a group of mothers while watching their own child engaged in a stressful situation with that of another group of women observing unknown children in the same situation. According to the exiting literature, reporting increased behavioral responses and higher neural activation in mothers watching their own distressed child, we expected that the two groups would have presented differences with respect to either the intensity and/or the timing of their physiological reactions. Our results indicate that, for both groups of women, the considered autonomic response significantly changed during the experimental condition with respect to the neutral one. The emotional attunement resulted slightly stronger and much faster in the mother-child group than in the other women-child group.

Specifically, according to the hypothesis that the emotional attunement with another person embodies a direct sharing of visceral-autonomic responses (Konvalinka et al., 2011), we found that the time course of the nose tip temperature confirmed that adult women shared such an autonomic response with child exposed to a distressful situation, independently of parenting relations with the child. When analyzing the single adult-child dyad response, the two groups of dyads differed. The autonomic responses stronger correlated during the experimental condition with respect to the neutral condition for all of the mothers-child dyads, while the same did not always happen in the other women group.

Moreover, the highest cross-correlation coefficient values between the time courses of the adult-child dyad signals during the experimental condition were found at a very short or null delay for the mother-child group, whereas a much longer delay characterized the other women-child group.

Group average correlation coefficient values did not significantly differ between the two groups. This result may rise an apparent discrepancy between the results found at a group level and at a single dyad level results. Such a mismatch likely depends on the procedure used to perform the group analysis, which emphasized the shared signals modulations within the dyads. As the experiment was designed to maximally preserve the natural and ecological context, there were no a priori limits or timing for the time duration of the phases' time duration. Therefore, it was not possible to adopt a unique common time frame the whole at the group level.

The last finding suggests that the emotional promptness to one's own child's distress is a distinct feature of maternal attitude, thus confirming at a physiological level the largely confirmed results found at a behavioral level (Stern, 1974, 1999; Brazelton et al., 1974; Beebe and Lachman, 1988; Tronick, 1989; Trevarthen, 1993; Fleming et al., 1999; Trevarthen and Aitken, 2001; Feldman, 2007; Feldman and Eidelman, 2007; Papousek, 2007; Feldman et al., 2012). Since such a faster promptness for emotional vicarious processes seems to characterize maternal responses, our results further suggest to consider it as a basic mechanism for organizing early mother-child interactions. The faster autonomic response of the mothers supports the idea that the Autonomic Nervous System plays a fundamental warning role for the mother with respect to the child's emotional shift (Frodi and Lamb, 1978; Gunnar and Donzella, 2002). This signal would modulate an embodied and pre-reflective sensitivity that helps the mother to immediately recognize any shift in the child's emotional needs, as well as to promptly soothe the child when distressed (Mills-Koonce et al., 2007).

The explorative, qualitative analysis within the other women group suggested interesting preliminary information about possible differences between mothers and not mothers looking unknown children. The small size of the sample does not allow drawing any conclusion, but our results suggest to investigate further whether the other mothers may have a stronger autonomic attunement with child and a faster response than non-mothers. Such a result would be in accordance with previous fMRI data showing different responses in brain regions involved in the attachment and in the integration of autonomic states with social behavior to infant negative emotions between the mothers and non-mothers (e.g., hypothalamic–midbrain–limbic–paralimbic–cortical circuits) (Seifritz et al., 2003; Feldman, 2007; Swain et al., 2008).

The present study crucially extends the results obtained by our previous work (Ebisch et al., 2012) where, using the same paradigm, we provided consistent evidence for a synchrony in emotional response of mothers watching their own distressed child. Following that evidence, the present study tested in a larger sample the hypothesis that the above synchrony was modulated by the bond between the woman and the child. Therefore, a control group of “other women” was recruited and compared with the “mother” group. A key improvement, from a methodological point of view, in the present work is represented by the use of a novel technique for extracting the thermal signal in pre-defined region of interest. The tracking algorithm allowed for an extraction of the signals that, despite the movements of the subjects, was objective over the entire experimental session, thus providing a stable and more accurate evaluation of temperature variations, together with an increased number of processed frames per condition. The extraordinary potentialities of thermal infrared imaging in this field of research have been further confirmed by this study, as we were able to record physiological measures of the vicarious reactions without interfering with the subjects' spontaneous behavior.

Our study present some limitations that should be addressed. Firstly, the sample size is somewhat small. Therefore, our results should be considered preliminary, but suggestive of new insights in the study of the body communication between adults and children. Larger sample would be desirable, even though it has to be recognized that is it difficult, and somehow complex, to involve young children with their parents in a physiological study on children's distress. However, we plan to enlarge the sample size. The choice of preserving the ecological context determined the needs for the rejection of a not irrelevant number of dyads because of the excessive movements of the child that caused a artifacts that our tracking software could not fix. In addition, it determined the impossibility for a obtaining a group average signal.

To conclude, the findings show that the child's distress evocates in the observing women a spontaneous autonomic response, which could reflect an emotional sharing ability, stronger and faster in the mothers. Therefore, our study supports the hypothesis that the maternal bond can modulate the promptness of the adult's response to the child's needs.

### Conflict of interest statement

The authors declare that the research was conducted in the absence of any commercial or financial relationships that could be construed as a potential conflict of interest.

## References

[B1] Anbar M. (2002). Assessment of physiologic and pathologic radiative heat dissipation using dynamic infrared imaging. Ann. N.Y. Acad. Sci. 972, 111–118 10.1111/j.1749-6632.2002.tb04560.x 12496005

[B2] Barrett C. K. (2005). The origins of social emotions and self-regulation in toddlerhood: new evidence. Cogn. Emot. 19, 973–979

[B3] Barrett C. K. Zahn-Waxler C. Cole P. M. (1993). Avoiders vs. Amenders: implications for the investigation of guilt and shame during toddler hood? Cogn. Motion 7, 481–505 10.1080/02699939308409201

[B4] Beebe B. Lachman F. M. (1988). The contribution of mother-infant mutual influence to the origins of self- and object representations. Psychoanal. Psychol. 5, 305–337 10.1037/0736-9735.5.4.305

[B5] Bell S. M. Ainsworth M. D. (1972). Infant crying and maternal responsiveness. Child Dev. 43, 1171–1190 10.2307/1127506 4643768

[B6] Bowlby J. (1958). The nature of the child's tie to his mother. Int. J. Psychoana. 39, 1–23 13610508

[B7] Brazelton T. B. Koslowski B. Main M. L. Michael R. Leonard A. (1974). The Origins of Reciprocity: the Early Mother-infant Interaction. The Effect of the Infant on its Caregiver. Oxford: Wiley-Interscience

[B7a] Cole P. M. Barrett K. C. Zahn-Waxler C. (1992). Emotion displays in two-year-olds during mishaps. Child Dev. 63, 314–324 10.1111/j.1467-8624.1992.tb01629.x 1611936

[B9] Ebisch S. J. Aureli T. Bafunno D. Cardone D. Romani G. L. Merla A. (2012). Mother and child in synchrony: thermal facial imprints of autonomic contagion. Biol. Psychol. 89, 123–129 10.1016/j.biopsycho.2011.09.018 22001267

[B9a] Feldman R. (2007). On the origins of background emotions: from affect synchrony to symbolic expression. Emotion 7, 601–611 10.1037/1528-3542.7.3.601 17683216

[B10] Feldman R. Eidelman A. I. (2007). Maternal postpartum behaviour and the emergence of infant-mother and infant-father synchrony in preterm and full-term infants: the role of neonatal vagal tone. Dev. Psychobiol. 49, 290–302 10.1002/dev.20220 17380505

[B11] Feldman R. Gordon I. Zagoory-Sharon O. (2010). Maternal and paternal plasma, salivary, and urinary oxytocin and parent-infant synchrony: considering stress and affiliation components of human bonding. Dev. Sci. 14, 752–761 10.1111/j.1467-7687.2010.01021.x 21676095

[B12] Feldman R. Magori-Cohen R. Galili G. Singer M. Louzoun Y. (2011). Mother and infant coordinate heart rhythms through episodes of interaction synchrony. Infant Behav. Dev. 34, 569–577 10.1016/j.infbeh.2011.06.008 21767879

[B12a] Feldman R. Zagoory-Sharon O. Weisman O. Schneiderman I. Gordon I. Maoz R. (2012). Sensitive parenting is associated with plasma oxytocin and polymorphisms in the OXTR and CD38 genes. Biol. Psychiatry 72, 175–181 10.1016/j.biopsych.2011.12.025 22336563

[B13] Fleming A. S. O'Day D. H. Kraemer G. W. (1999). Neurobiology of mother-infant interactions: experience and central nervous system plasticity across development and generations. Neurosci. Biobehav. Rev. 23, 673–685 10.1016/S0149-7634(99)00011-1 10392659

[B14] Frodi A. M. Lamb M. E. (1978). Sex differences in responsiveness to infants: a developmental study of psychophysiological and behavioural responses. Child Dev. 49, 1182–1188 10.2307/1128758 738152

[B15] Gallese V. Keysers C. Rizzolatti G. (2004). A unifying view of the basis of social cognition. Trends Cogn. Sci. 8, 396–403 10.1016/j.tics.2004.07.002 15350240

[B16] Guedeney A. Guedeney N. Tereno S. Dugravier R. Greacen T. Welniarz B. (2011). Infant rhythms versus parental time: promoting parent-infant synchrony. J. Physiol. Paris 105, 195–200 10.1016/j.jphysparis.2011.07.005 21782020

[B17] Gunnar R. M. Donzella B. (2002). Social regulation of the cortisol levels in early human development. Psychoneuroendocrinology 27, 199–220 10.1016/S0306-4530(01)00045-2 11750779

[B18] Haley D. W. Stansbury K. (2003). Infant stress and parent responsiveness: regulation of physiology and behaviour during still-face and reunion. Child Dev. 74, 1534–1546 10.1111/1467-8624.00621 14552412

[B19] Iacoboni M. Woods R. P. Brass M. Bekkering H. Mazziotta J. C. Rizzolatti G. (1999). Cortical mechanisms of human imitation. Science 286, 2526–2528 10.1126/science.286.5449.2526 10617472

[B20] Kim P. Leckman J. F. Mayes L. C. Feldman R. Wang X. Swain J. E. (2010). The plasticity of human maternal brain: longitudinal changes in brain anatomy during the early postpartum period. Behav. Neurosci. 124, 695–700 10.1037/a0020884 20939669 PMC4318549

[B21] Kistler A. Mariauzouls C. von Berlepsch K. (1998). Fingertip temperature as an indicator for symphatetic responses. Int. J. Psychopatol. 29, 35–41 10.1016/S0167-8760(97)00087-1 9641246

[B22] Kochanska G. Aksan N. Koenig A. L. (1995). A longitudinal study of the roots of preschoolers' conscience: committed compliance and emerging internalization. Child Dev. 66, 1752–1769 10.2307/1131908 8556897

[B23] Kochanska G. Gross J. N. Lin M. H. Nichols K. E. (2002). Guilt in young children: development, determinants, and relations with a broader system of standards. Child Dev. 73, 461–482 10.1111/1467-8624.00418 11949903

[B24] Konvalinka I. Xygalatasa D. Bulbuliac J. Schjødta U. Jegindøa E. M. Wallotd S. (2011). Synchronized arousal between performers and related spectators in a fire-walking ritual. Proc. Natl. Acad. Sci. U.S.A. 2, 8514–8519 10.1073/pnas.1016955108 21536887 PMC3100954

[B25] Kuraoka K. Nakamura K. (2011). The use of nasal skin temperature measurements in studying emotion in macaque monkeys. Physiol. Behav. 102, 347–355 10.1016/j.physbeh.2010.11.029 21130103

[B26] Lenzi D. Trentini C. Pantano P. Macaluso E. Iacoboni M. Lenzi G. L. (2008). Neural basis of maternal communication and emotional expression processing during infant preverbal stage. Cereb. Cortex 19, 1124–1133 10.1093/cercor/bhn153 18787229

[B26a] Lewis M. Sullivan M. W. Stanger C. Weiss M. (1998). Self development and self conscious emotions. Child Dev. 60, 146–156 2702864

[B27] Merla A. Romani G. L. (2007). Thermal signatures of emotional arousal: a functional infrared imaging study. Conf. Proc. IEEE Eng. Med. Biol. Soc. 2007, 247–249 10.1109/IEMBS.2007.4352270 18001936

[B27a] Mills R. S. L. (2003). Possible antecedents and developmental implications of shame in young girls. Inf. Child Dev. 12, 329–349 10.1002/icd.308

[B28] Mills-Koonce W. R. Gariépy J. L. Propper C. Sutton K. Calkins S. Moore G. (2007). Infant and parent factors associated with early maternal sensitivity: a caregiver-attachment systems approach. Infant Behav. Dev. 30, 114–126 10.1016/j.infbeh.2006.11.010 17292784

[B29] Mogi K. Nagasawa M. Kikusui T. (2011). Developmental consequences and biological significance of mother-infant bonding. Prog. Neuropsychopharmacol. Biol. Psychiatry 35, 1232–1241 10.1016/j.pnpbp.2010.08.024 20817069

[B30] Nakanishi R. Imai-Matsumura K. (2008). Facial skin temperature decreases in infants with joyful expression. Infant Behav. Dev. 31, 137–144 10.1016/j.infbeh.2007.09.001 17983661

[B31] Nhan B. R. Chau T. (2010). Classifying affective states using thermal infrared imaging of the human face. IEEE Trans. Biomed. Eng. 57, 979–987 10.1109/TBME.2009.2035926 19923040

[B32] Noriuchi M. Kikuchi Y. Senoo A. (2008). The functional neuroanatomy of maternal love: mother's response to infant's attachment behaviours. Biol. Psychiatry 63, 415–423 10.1016/j.biopsych.2007.05.018 17686467

[B32a] Papousek M. (2007). Communication in early infancy: an arena of intersubjective learning. Infant Behav. Dev. 30, 258–266 10.1016/j.infbeh.2007.02.003 17363062

[B33] Porges S. W. (1998). Love: an emergent property of the mammalian autonomic nervous system. Psychoneuroendocrinology 23, 837–861 10.1016/S0306-4530(98)00057-2 9924740

[B34] Porges S. W. (2003a). Social engagement and attachment: a phylogenetic perspective. Ann. N.Y. Acad. Sci. 1008, 31–47 10.1196/annals.1301.004 14998870

[B35] Porges S. W. (2003b). The polyvagal theory: phylogenetic contributions to social behaviour. Physiol. Behav. 79, 503–513 10.1016/S0031-9384(03)00156-2 12954445

[B36] Rizzolatti G. Fogassi L. Gallese V. (2001). Neurophysiological mechanisms underlying the understanding and imitation of action. Nat. Rev. Neurosci. 2, 661–670 10.1038/35090060 11533734

[B37] Roddie I. (1963). The role of vasoconstrictor and vasodilator nerves to skin and muscle in the regulation of the human circulation. Ann. R. Coll. Surg. Engl. 32, 180–193 13974599 PMC2311538

[B38] Seifritz E. Esposito F. Neuhoff J. G. Lüthi A. Mustovic H. Dammann G. (2003). Differential sex-independent amygdala response to infant crying and laughing in parents versus nonparents. Biol. Psychiatry 54, 1367–1375 10.1016/S0006-3223(03)00697-8 14675800

[B39] Sethre-Hofstad L. Stansbury K. Rice M. A. (2002). Attunement of maternal and child adrenocortical response to child challenge. Psychoneuroendocrinology 27, 731–747 10.1016/S0306-4530(01)00077-4 12084665

[B40] Shastri D. Merla A. Tsiamyrtzis P. Pavlidis I. (2009). Imaging facial signs of neurophysiological responses. IEEE Trans. Biomed. Eng. 56, 477–484 10.1109/TBME.2008.2003265 19272941

[B41] Stern D. (1985). World of the Infant: A View from Psychoanalysis and Developmental Psychology. New York, NY: Karnac Book

[B41a] Stern D. N. (1974). Mother and infant at play: The dyadic interaction involving facial, vocal, and gaze behaviors, in The Effect of the Infant on Its Caregiver, XXIV, eds Lewis R. M. Rosenblum L. A. (Oxford: Wiley-Interscience), 264

[B41b] Stern D. N. (1999). Vitality contours: The temporal contour of feelings as a basic unit for constructing the infant's social experience, in Early Social Cognition: Understanding Others in the First Months of Life, ed Rochat P. (Mahwah, NJ: Lawrence Erlbaum Associates Publishers), 67–80

[B41aa] Stipek D. (1995). The development of pride and shame in toddlers, in Self-Conscious Emotions: The Psychology of Shame, Guilt, Embarrassment, and Pride, XVII, eds Tangney J. P. Fischer K. W. (New York, NY: Guilford Press), 237–252

[B42] Sullivan R. Perry R. Sloan A. Kleinhaus K. Burtchen N. (2011). Infant bonding and attachment to the caregiver: insights from basic and clinical science. Clin. Perinatol. 38, 643–655 10.1016/j.clp.2011.08.011 22107895 PMC3223373

[B43] Swain J. E. (2011). The human parental brain: *in vivo* neuroimaging. Prog. Neuropsychopharmacol. Biol. Psychiatry 35, 1242–1254 10.1016/j.pnpbp.2010.10.017 21036196 PMC4329016

[B44] Swain J. E. Kim P. Ho S. S. (2011). Neuroendocrinology of parental response to baby-cry. J. Neuroendocrinol. 11, 1036–1041 10.1111/j.1365-2826.2011.02212.x 21848646 PMC4319977

[B45] Swain J. E. Lorberbaum J. P. Kose S. Strathearn L. (2007). Brain basis of early parent-infant interactions: psychology, physiology, and *in vivo* functional neuroimaging studies. J. Child Psychol. Psychiatry 48, 262–287 10.1111/j.1469-7610.2007.01731.x 17355399 PMC4318551

[B46] Swain J. E. Tasgin E. Mayes L. C. Feldman R. Constable R. T. Leckman J. F. (2008). Maternal brain response to own baby-cry is affected by cesarean section delivery. J. Child Psychol. Psychiatry 49, 1042–1052 10.1111/j.1469-7610.2008.01963.x 18771508 PMC3246837

[B47] Tangherlini A. Merla A. Romani G. L. (2006). Field-warp registration for biomedical high-resolution thermal infrared images. Conf. Proc. IEEE Eng. Med. Biol. Soc. 1, 961–964 10.1109/IEMBS.2006.260664 17946013

[B47a] Tangney J. P. Fischer K. W. (1995). Self-Conscious Emotions. The Psychology of Shame, Guilt, Embarrassment, and Pride. New York, NY: Guilford Press

[B48] Trevarthen C. Aitken K. J. (2001). Infant intersubjectivity: research, theory, and clinical applications. J. Child Psychol. Psychiatry 42, 3–48 10.1111/1469-7610.00701 11205623

[B48a] Trevarthen C. (1993). The self born in intersubjectivity: the psychology of an infant communicating, in The Perceived Self: Ecological and Interpersonal Sources of Self-Knowledge. Emory Symposia in Cognition, Vol. 5, ed Neisser U. (New York, NY: Cambridge University Press), 121–173

[B49] Tronick E. Z. (1989). Emotions and emotional communications in infants. Am. Psychol. 44, 112–119 10.1037/0003-066X.44.2.112 2653124

